# Combinatorial Effect of ARTP Mutagenesis and Ribosome Engineering on an Industrial Strain of *Streptomyces albus* S12 for Enhanced Biosynthesis of Salinomycin

**DOI:** 10.3389/fbioe.2019.00212

**Published:** 2019-09-03

**Authors:** Kuipu Zhang, Ali Mohsin, Yichen Dai, Zhongbing Chen, Yingping Zhuang, Ju Chu, Meijin Guo

**Affiliations:** ^1^State Key Laboratory of Bioreactor Engineering, East China University of Science and Technology, Shanghai, China; ^2^Zhejiang Biok Biology Co., Ltd., Zhongguan Industrial Park, Zhejiang, China

**Keywords:** agar block diffusion method, atmospheric and room temperature plasma, ribosome engineering, transcriptome analysis, salinomycin, glyoxylate metabolism

## Abstract

Salinomycin, an important polyketide, has been widely utilized in agriculture to inhibit growth of pathogenic bacteria. In addition, salinomycin has great potential in treatment of cancer cells. Due to inherited characteristics and beneficial potential, its demand is also inclining. Therefore, there is an urgent need to increase the current high demand of salinomycin. In order to obtain a high-yield mutant strain of salinomycin, the present work has developed an efficient breeding process of *Streptomyces albus* by using atmospheric and room temperature plasma (ARTP) combined with ribosome engineering. In this study, we investigate the presented method as it has the advantage of significantly shortening mutant screening duration by using an agar block diffusion method, as compared to other traditional strain breeding methods. As a result, the obtained mutant Tet^30^Chl^25^ with tetracycline and chloramphenicol resistance provided a salinomycin yield of 34,712 mg/L in shake flask culture, which was over 2.0-fold the parental strain S12. In addition, comparative transcriptome analysis of low and high yield mutants, and a parental strain revealed the mechanistic insight of biosynthesis pathways, in which metabolic pathways including butanoate metabolism, starch and sucrose metabolism and glyoxylate metabolism were closely associated with salinomycin biosynthesis. Moreover, we also confirmed that enhanced flux of glyoxylate metabolism via overexpression gene of isocitrate lyase (*icl*) promoted salinomycin biosynthesis. Based on these results, it has been successfully verified that the overexpression of crotonyl-CoA reductase gene (*crr*) and transcriptional regulator genes (*orf* 3 and *orf* 15), located in salinomycin synthesis gene cluster, is possibly responsible for the increase in salinomycin production in a typical strain *Streptomyces albus* DSM41398. Conclusively, a tentative regulatory model of ribosome engineering combined with ARTP in *S. ablus* is proposed to explore the roles of transcriptional regulators and stringent responses in the biosynthesis regulation of salinomycin.

## Introduction

Salinomycin, being one of polyether antibiotics, is largely produced by *Streptomyces albus*. It has been widely used in animal husbandry as a growth promoter for over 30 years (Gumila et al., 1997). Besides, it has been recently proven as a good potential agent to inhibit leukemia stem cells (Fuchs et al., 2010) and epithelial cancer stem cells (Kuo et al., 2012). The gene cluster of salinomycin biosynthesis is about a fragment of 126 kb, which consists of nine type-I polyketide synthases (PKSs) (*slnA1* to *slnA9*) and five genes encoding transcription regulators: *orf3* (*SLNWT_0290*, transcription regulator AsnC), *orf9* (*SLNWT_0284*, transcription regulator), *orf10* (*SLNWT_0283*, transcription regulator TrmB), *orf15* (*SLNWT_0255*, SARP family transcription regulator), and *slnR* (*SLNWT_0261*, LuxR family transcription regulator). Among these, SlnR has been proven as a positive pathway-specific regulator. However, the exact functions of *orf3, orf9, orf10, orf15* are still not clear (Jiang et al., 2012; Zhu et al., 2017).

Nowadays, a hot topic for researchers is to find out possible routes for improving the ability of *Streptomyces* to produce high-level antibiotics. Especially when wild-type strains isolated from nature only produce a low level (1–100 ug/mL) of antibiotics, which is failing to meet high commercial requirements (Luhavaya et al., 2015). As a result, a great deal of effort and resources are being undertaken to improve antibiotic-yield and have been for decades. Current methods that are generally used to enhance productivity of industrial microorganisms are classical random approaches and highly rational methods, such as metabolic engineering or genetic manipulation. However, regarding industrial strains producing secondary metabolites like antibiotics, classical methods are still considered effective even without using genomic information or genetic tools (Zhu et al., 2006).

During recent years, atmospheric and room temperature plasma (ARTP) has been considered as one of the most effective and novel types of microbial mutation breeding technology. The core component of ARTP is RF APGD plasma generator, which generates a plasma jet that can change structure and permeability of the cell wall and plasma membrane, leading to DNA damage, including missense mutation, deletion or nucleotide frameshift mutation (Fang et al., 2013; Li et al., 2014; Zhang et al., 2014). However, the most important parameters of an ARTP system, such as helium gas flow rate (standard liter per minute, SLPM), gap space between nozzle and sample plate (millimeter, mm), energy input (Watt, W), and treatment time (seconds, s), need to be investigated for optimization of plasma mutation conditions. Although ARTP has several outstanding advantages over other conventional methods, including rapid mutation rate and high operational flexibility (Zhang et al., 2014). In order to obtain a desired target, a single ARTP treatment or an iterative ARTP treatment is usually performed (Wang et al., 2010; Zhang et al., 2015; Ren et al., 2017; Cao et al., 2018). Interestingly, in previous studies, ARTP treatment was also combined with other treatments such as single resistance screening (Liang et al., 2015; Ren et al., 2017), or radiation mutagenesis with UV (Zhang et al., 2015) to improve the overall efficiency.

Apart from this, ARTP has been extensively used in several applications relating to microalgaes, bacteria, fungi, yeasts, and actinomycetes (Zhang et al., 2014). Cao et al. (2017) bred one mutant II-H6 of *Chlorella pyrenoidosa* by ARTP Compared with the original strain, the biomass and lipid productivity of II-H6 increased by 22.07 and 16.85%, respectively. Similarly, Dong et al. (2016) utilized ARTP mutagenesis to enhance substrate tolerance of *Pseudomonas putida* and obtained a 42% increase in nicotinic acid yield mutant. Additionally, this system has been successfully applied in *Streptomyces* species, such as *Streptomyces avermitilis* and *Streptomyces bingchenggensis*, to improve avermectin and milbemycins A3/A4 production levels, respectively (Wang et al., 2010, 2014). Therefore, ARTP is considered to be one of the most efficient techniques for enhancing productivity of desired target product.

On the other hand, ribosome engineering technique has been largely applied to activate the potential ability of bacteria to produce various important secondary metabolites. Generally, antibiotics such as streptomycin, tetracycline, kanamycin, and chloramphenicol have the ability to bind with ribosomal proteins, which resulted in mutations of ribosomal proteins (Tamehiro et al., 2003). Moreover, when strains are treated by antibiotics, the survived variants faced random mutations in their ribosomal proteins or RNA polymerase components, followed by changes in gene expressions at transcriptional level, which probably led to a significant improvement in secondary metabolite synthesis in mutants (Shima et al., 1996; Hosoya et al., 1998; Hosaka et al., 2006; Kurosawa et al., 2006). Previous studies have shown that ribosome engineering and stringent response were closely linked in the regulation of secondary metabolism of cells (Ochi et al., 2003). Furthermore, ribosomal proteins, pentaphosphate/tetraphosphate ((p)ppGpp), and RNA polymerases were considered crucial for the regulation of secondary metabolism. Particularly, (p)ppGpp is suggested to play a central role in triggering the initial steps of antibiotic production in *Streptomyces*. According to one of the previous studies, deletion of gene *relA* (related to synthesis of (p)ppGpp)) led to an impairment on antibiotic production (Jin et al., 2004).

Based on the literature and facts, picking out high-yield antibiotic production mutants from a rich mutant library of thousands of strains by 24-well plates fermentation is laborious work. Therefore, a rapid and effective screening method is necessary to obtain target mutants. For salinomycin producer strain, it generally takes over 20 days for one round from spore preparation to mutants' selection. For this purpose, the present work aims to develop an efficient breeding screening method for high-yield salinomycin biosynthesis mutants. Herein, ARTP and ribosome engineering are integrated together in prescreening of high-yield mutants of salinomycin, followed by an agar block diffusion screening, which aided fast screening of high salinomycin titer mutants based on inhibition zone (Figure S1) observed on the culture plates. Meanwhile, we elucidate the logical reasoning for the improved performance of salinomycin biosynthesis mutants based on transcriptomic data analysis of mutants and parental strain. Furthermore, a tentative regulatory model of antibiotic resistance screening and ARTP mutagenesis in *S. albus* is proposed.

## Materials and Methods

### Strains and Culture Conditions

*Streptomyces albus* S12 and their derivatives were grown on ISP4 agar plates (BD, USA) for sporulation. For shake flask fermentation, 100 μL of spore suspension was inoculated in a 250 mL flask containing 30 mL fresh medium (4.0% glucose, 3.0% soy flour, 1.0% yeast extract, 0.2% calcium carbonate) and further incubated for 26–32 h as a seed culture medium. After that, 3 mL of seed culture was transferred into 30 mL of fermentation medium (0.8% germ powder, 0.5% soybean powder, 0.2% potassium chloride, 0.10% sodium chloride, 0.16% urea, 0.2% tartaric acid, 0.01% magnesium sulfate, 0.01% dibasic potassium phosphate, 0.5% calcium carbonate, 15% soybean oil, pH 6.6–6.9) and then incubated for 7 days in a rotary shaker at 33°C with shaking speed of 250 rpm.

### Random Mutagenesis by ARTP

An automated ARTP mutation breeding system (Siqingyuan Biotechnology Co., Ltd., Wuxi, China) was utilized in this study. The main parameters include: power source P (P = 100 W), gas flow G (G = 10 slm), distance between emission source and carrier D (D = 2.5 mm), and temperature of plasma T (T = 28°C) (Fang et al., 2013). Ten microliter of fresh spore suspension (10^6^-10^8^ cells/mL) was evenly spread on a sterilized metal slide and exposed to ARTP jet for 0, 60, 120, 180, 240, 300, 360, 420, 480, 540, and 600 s, respectively. Based on the results shown in Figure S2, the exposure time employed in this study was set at 360 s to obtain a desirable lethality rate. After each treatment, the slides were transferred to sterilized EP tubes containing 1.0 mL of fermentation medium and then incubated in a shaking incubator at 30°C and 120 rpm for 3 h. At last, 100 μL of the diluent was cultivated on ISP4 solid medium for 8 days at 30°C.

### Resistance Screening (Two Rounds)

**Figure 1:**
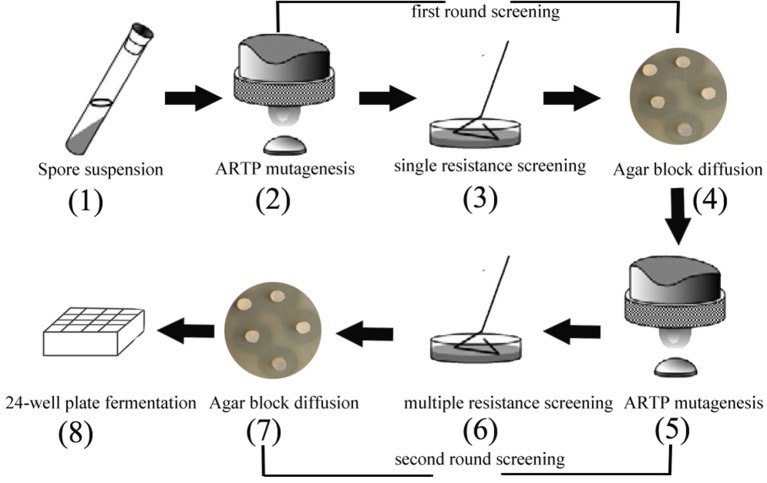
The two rounds of resistance screening in preliminary screening.

The minimum inhibitory concentration (MIC) of kanamycin (Kan) was 1.0 μg/mL, of tetracycline (Tet) was 20 μg/mL, and of chloramphenicol (Chl) was 15 μg/mL (data not shown). According to results of the minimum inhibitory concentration, the antibiotic concentrations were designed as adaptive pressure, as shown in Tables S1–S5. The suspension from spores were exposed to two rounds of resistance screening (Figure 1). The first round of drug resistance screening was single resistance screening (Kan, Chl, Tet), which started from step 2 to step 4. After that, the second round of resistance screening was multiple resistance screening (Kan-Chl, Kan-Tet, Chl-Tet, Kan-Chl-Tet), which started from step 5 to step 7. In this process, after treating by the first round of resistance screening, the selected spores were mixed and then exposed to a second round of resistance screening.

### Agar Block Diffusion Method

A loop of *B. pumilus* grown on a slant was inoculated into a test tube containing 5.0 mL fresh Luria-Bertani medium (1.0% NaCl, 0.5% yeast extract, 1.0% tryptone) and grown for 24 h at 33°C with shaking speed of 220 rpm. After that, 1.0 mL of cell suspension was centrifuged at 12,000 rpm for 5 min. Then, supernatant was discarded, pellets were washed by 500 μL sterilized water once and re-suspended by 500 μL of sterilized 0.85% NaCl solution. Subsequently, 100 μL of re-suspended spores of *B. pumilus* was added into a LB agar plate. *S. albus* S12 and derivatives were grown on ISP4 agar plates (BD, USA) for sporulation. 1 cm^2^ agar blocks of spores were picked after 5 days of culturing and then co-cultured with *B. pumilus* cells at 33°C for 24 h on LB agar plates.

### High-Throughput Screening

For 24 deep-well plates fermentation, spore suspension was inoculated into 24 deep-well plates containing 1.2 mL of seed culture medium (4.0% glucose, 3.0% soybean flour, 1.0% yeast extract, 0.2% calcium carbonate) and grown at 33 °C with shaking speed of 250 rpm for 26 h. After that, 120 μL of seed culture was transferred into a 24 deep-well plate containing 1.2 mL fermentation medium (0.8% germ powder, 0.5% soybean powder, 0.2% potassium chloride, 0.10% sodium chloride, 0.16% urea, 0.2% tartaric acid, 0.01% magnesium sulfate, 0.01% dibasic potassium phosphate, 0.5% calcium carbonate, and 15% soybean oil, pH 6.6–6.9) on a rotary shaker at 250 rpm and 33 °C for 7 days. Each experiment was performed in triplicates.

### RNA Extraction and RNA-Sequencing

RNA samples from the mutant strain TK, Tet^30^Chl^25^, and the parental strain, S12, were analyzed at the 5th day of fermentation. After that, these samples were directly frozen using liquid nitrogen and then stored at −80°C.

Sequencing and subsequent bioinformatics analysis were completed at Novel Bioinformatics Co., Ltd, Shanghai, China. Total RNA was extracted with Trizol reagent (Invitrogen, Grand Island, NY, USA). RNA quality was checked by capillary electrophoresis (Bioanalyzer 2200, Aligent, Santa Clara, USA). Ribosomal RNA was depleted with Ribo-Zero^TM^ rRNA Removal Kits (Illumina Inc., San Diego, CA), and cDNA library construction was carried out using the TruSeq Stranded mRNA Library Preparation Kit (Illumina). Raw data are available on the Gene Expression Omnibus database (accession GSE133580).

### Overexpression of Genes Under the Control of PermE^*^ Promoter in *S. albus* DSM41398

The putative *ccr* gene sequence was obtained from genome sequence of *S. albus* DSM41398 (https://www.ncbi.nlm.nih.gov/nuccore/NZ_CP010519.1). The *ccr* gene used for overexpression was amplified with primers pair *ccr*-F-*Nde*I and *ccr*-R-*Eco*RI, and the product was cloned to pMD18-T and verified via sequencing. A *Nde*I-*Eco*RI fragment with *ccr* gene was ligated with *Nde*I-*Eco*RI-digested plasmid pIB139 to generate pIB-*crr*. Then an *Eco*RI fragment containing apramycin resistance gene *aac(3)IV* was ligated with *Eco*RI-digested pIB-crr. The resulted plasmid was introduced into wild-type competent cells through conjugation, and apramycin-resistant conjugants were selected and further verified by PCR.

Other over-expressed genes were also similarly performed.

### Determination of Salinomycin Production in *Streptomyces albus*

Determination of salinomycin production was conducted by following procedures described previously (Lu et al., 2016). Fermentation cultures of wild-type strain and mutants were extracted with 9 volumes of methanol, then ultrasonic extraction for 30 min. Samples were applied to liquid chromatography after passing through 0.2 μm filters. The liquid chromatograph was operated at a flow rate of 1 mL/min with an Agilent Eclipse TC-C_18_ column (4.6 by 250 mm; particle size, 5 μm) with UV detection at 210 nm. The column temperature was set at 25°C, and the mobile phase was Merck HPLC grade acetonitrile-2% aqueous acetic acid (92:8 [v/v]), and the injection volume was 20 μL.

### Electrophoretic Mobility Shift Assay (EMSA)

The binding sites were supposed to locate in the upstream region (from −300 bp to +50 bp relative to translational start site of target genes), primers were designed and attached with a universal primer (5′-AGCCAGTGGCGATAAG-3′), the PCR products were biotin-labeled by PCR with 5′ biotin-modified universal primer. The concentrations of PCR products were determined by Nanodrop2000 (Thermo Scientific, Germany). EMSAs were performed as per the instructions by Chemiluminescent EMSA Kit (Beyotime Biotechnology, China). The binding reaction contained 10 mM Tris-HCl pH 8.0, 25 mM MgCl_2_, 50 mM NaCl, 1 mM DTT, 1 mM EDTA, 0.01% Nonidet P40, 50 mg/L poly[d(I-C)], 10% glycerol. After binding, samples were separated on a non-denatured PAGE gel in ice-bathed at 100 V for 40 min and bands were detected by BeyoECL Plus after dyeing (Beyotime Biotechnology, China).

## Results

### Development of Efficient Breeding Method for High-Yield Salinomycin Producer Mutant

**Figure 2:**
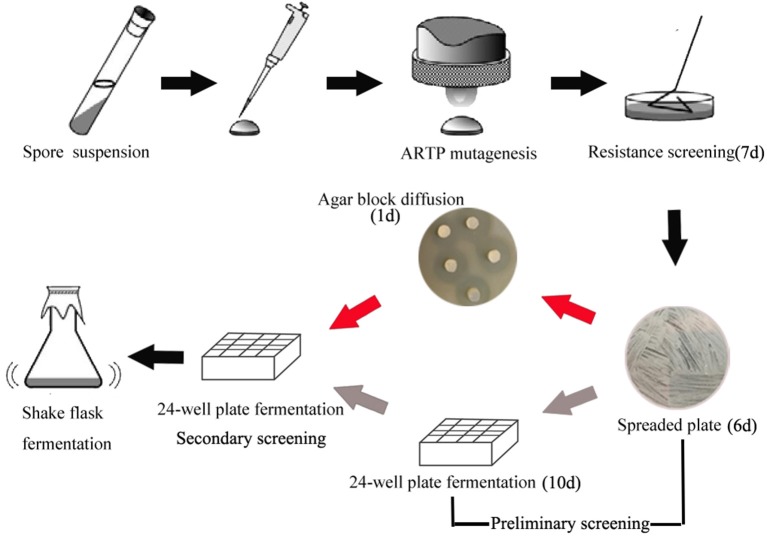
Optimized breeding method screening procedure. The gray arrow in preliminary represent the original breeding process, the red arrow in preliminary represent the developed breeding process.

In order to develop an effective breeding process of *S. albus* for improved biosynthesis of salinomycin, ARTP mutagenesis, antibiotic resistance screening and high-throughput screening (HTS) were comprehensively applied for efficient screening of high-yield strains of salinomycin. The whole breeding process is shown in Figure 2. Compared with original breeding process, 24-well plate fermentation screening in preliminary steps was replaced by a fast test method called agar block diffusion method. This method was based on the fact that there was a very close correlation between diameter of inhibitory zone and titer of salinomycin, which shows that the value of correlation coefficient r reached to 0.9559 (Figure S4). For the best case, the inhibition zone for measurement should be clear and well-bounded, therefore, we investigated the effect of optical density of indicator bacteria *Bacillus pumilus*, as well as the spores population of *Streptomyces albus* on diameter of inhibitory zone. As shown in Table S6 and Figure S3, spores of *Streptomyces albus* cultivated on ISP4 agar plates for 5 days, and co-cultivated with 0.1% *Bacillus pumilus*, could obtain a desirable inhibition zone. The single colony, which is capable of generating a larger diameter of inhibition zone than that produced by the parental strain S12 was selected for the next round of screening. As shown in Figure 2, it took almost 10 days to pick out target mutants for one round screening by 24-well plate fermentation screening. Comparatively, only 1 day is required when using agar block diffusion method. As a result, a total of 18 days were reduced in the entire process of two round screening, which greatly improves the efficiency of the strain breeding process.

### Screening of High-Yield Mutants of Salinomycin by ARTP and Ribosome Engineering

The treatment time of spores is very crucial for ARTP mutagenesis to obtain a desirable mutant rate. As shown in Figure S2, spores of *Streptomyces albus* were extremely sensitive to the mutagenesis dose when the treatment time was 600 s. Thus, no spores survived at 600 s. Considering the efficiency of mutation and selection processes, 360 s of treatment time was chosen as the sub-lethal dose. After being treated by APTP mutagenesis, the spore solution was coated on a ISP4 plate containing the corresponding resistance.

**Figure 3:**
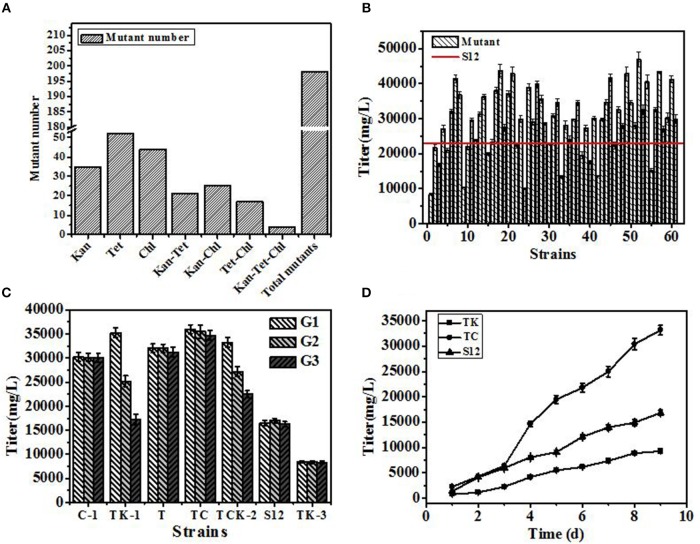
The results of the developed breeding process. **(A)** Number of mutants selected in resistance screening. **(B)** Titer of salinomycin production by mutants grown in shaking flask culture. **(C)** Genetic stability of selected mutants after 3 generations. **(D)** Titer of salinomycin production by mutants TK, Tet^30^Chl^25^, and the initial strain S12 grown in shaking flask culture. Error bars indicate standard error of the mean.

Mutants derived from drug resistance screening are shown in Figure 3A. In single resistance screening, 131 mutants (Kan 35, Chl 44, Tet 52) were selected separately, while in multiple resistance screening 67 mutants (Kan-Chl 25, Kan-Tet 21, Chl-Tet 17, Kan-Chl-Tet 4) were procured, respectively. Therefore, a total of 198 mutants were selected by resistance screening. In the following method of agar block screening, 61 out of 198 mutants with larger diameters of inhibition zone than that produced by the original strain S12 were selected. Later, 61 positive mutants were fermented in 24-well plates with the initial strain S12 as a control to check the accuracy of agar block method screening. As shown in Figure 3B, over 70 percent (35 out of 61) of mutants produced a higher salinomycin titer than control strain S12, and a positive mutation rate (R_P_) (35 out of 198) reached 17.7%, indicating that the agar block method could precisely select high-yield salinomycin mutants during preliminary screening.

Genetic stability of 5 mutants (named C-1, TK-1, T, Tet^30^Chl^25^, TCK-2), with a titer of salinomycin production >35,000 mg/L, and a negative mutant TK-3 were further investigated. The highest salinomycin production mutant Tet^30^Chl^25^ and the lowest salinomycin production mutant TK-3(named TK afterward) were finally obtained based on genetic stability (Figure 3C).

To further study the differences in salinomycin synthesis ability of TK, Tet^30^Chl^25^, and S12, shake flask fermentation was carried out, and samples were taken after every 24 h for 9 days. As shown in Figure 3D, compared with the initial strain S12, mutant strain Tet^30^Chl^25^ showed a 2-fold increase in production of salinomycin, while negative mutant strain TK showed a 44% decrease in production of salinomycin.

### Gene Expression Pattern Analysis for Mutants Grown in Shaking Flask Culture

The differences among mutant strain Tet^30^Chl^25^ (a positive mutant with high yield of salinomycin), TK (a negative mutant with low yield of salinomycin) and the parental industrial strain S12 transcriptomes were analyzed at day 5 of fermentation. As shown in Figure S5, a total of 1602 genes showed differences in expression between mutant strain TK, Tet^30^Chl^25^, and the initial strain S12. Transcriptomes of mutant strain TK, Tet^30^Chl^25^, and the initial strain S12 were compared, respectively. For S12 vs. Tet^30^Chl^25^, 937 transcripts were down-regulated in Tet^30^Chl^25^ mutant as compared with the initial strain S12, 16 transcripts were up-regulated, and 6377 transcripts did not show significant variations (Figure S5A).

KEGG pathway analysis of differentially expressed genes (DEGs) between mutant strain TK, Tet^30^Chl^25^, and the initial strain S12 were analyzed (Table 1). Ribosomal structure and biogenesis, mainly related to protein synthesis, showed an obvious up-regulation, and carbohydrate transport and metabolism, which may affect energy metabolism, showed a down-regulation in Tet^30^Chl^25^ mutant as compared with the initial strain S12. This indicated that high-yield salinomycin production mutant may exhibit enhanced protein synthesis activity and possessed a lower flux of energy producing pathway. These results are in agreement with the previous study, which reported that the K88E rpsL mutant of *S. coelicolor* showed enhanced Act production, exhibiting unusual protein synthesis activity (Hosaka et al., 2004). For TK vs. S12, aminobenzoate degradation and glyoxylate and dicarboxylate metabolism showed significant down regulation in TK strain, whereas, no pathway showed significant up-regulation.

**Table 1:** KEGG pathway analysis of genes differentially expressed genes (DEGs) between the mutant strain TK, Tet^30^Chl^25^, and the initial strain S12.

**Category**	**Pathway term**	***P*-value**	**(–log2P)**
Tet^30^Chl^25^ vs. S12 (up-regulated)	Translation, ribosomal structure and biogenesis	0.001	8.991
	Amino acid transport and metabolism	0.155	2.685
	Inorganic ion transport and metabolism	0.184	2.438
Tet^30^Chl^25^ vs. S12 (down-regulated)	Carbohydrate transport and metabolism	7.36E-09	27.016
	Beta-Alanine metabolism	0.0085	6.867
	Carotenoid biosynthesis	0.0168	5.888
TK vs. S12 (up-regulated)	Defense mechanisms	0.12	2.96
	General function prediction only	0.310	1.68
TK vs. S12 (down-regulated)	Aminobenzoate degradation	9.553E-06	16.675
	Glyoxylate and dicarboxylate metabolism	0.0125	6.319
	Amino sugar and nucleotide sugar metabolism	0.052	4.253
	Folate biosynthesis	0.052	4.241

**Figure 4:**
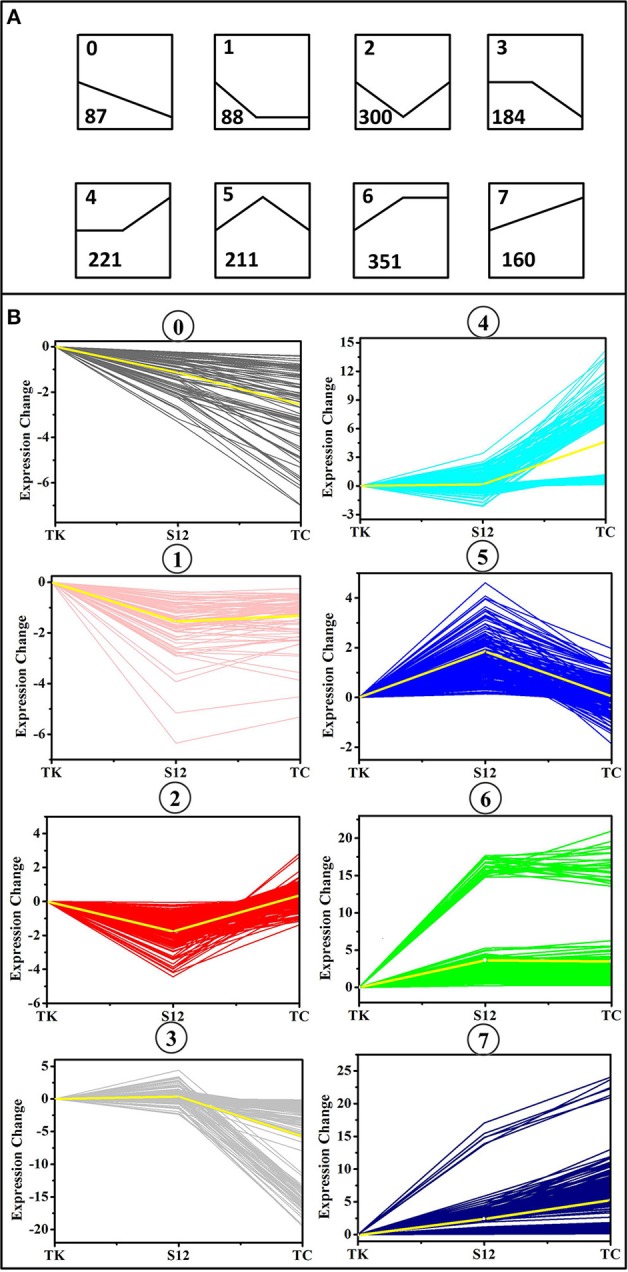
Clusters of differentially expressed genes (DEGs) between the mutant strain TK, Tet^30^Chl^25^, and the initial strain S12. **(A)** Eight trend models used by gene clustering analysis. The number in the upper left corner of each trend represents the type number to which the trend belongs, and the number in the lower left corner represents the number of genes that fall into the trend. **(B)** Clusters of differentially expressed genes (DEGs) under various salinomycin production environment. The X axis represents mutants TK, Tet^30^Chl^25^, and the parental strain S12 respectively.

**Figure 5:**
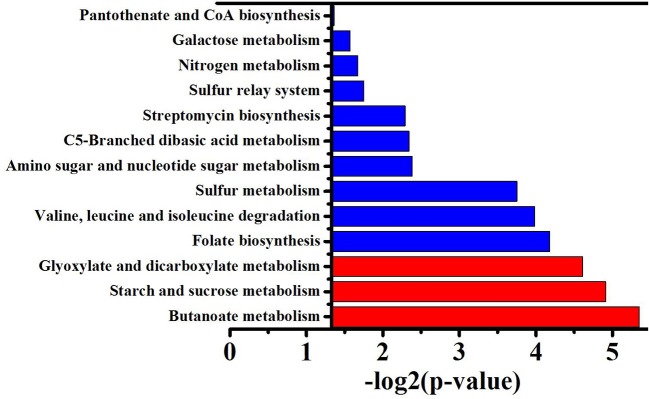
GO function enrichment analysis of genes from the cluster 7. *P*-value represents the level of signifiance, and treated by –log_2_, the smaller the *p*-value, the larger –log_2_P, and the better the significance. Red column means correlation is significant, blue column means not significant.

To obtain the main metabolic characteristics of *S. albus* grown under different conditions with respect to salinomycin production, the comparison of mutant strain TK, Tet^30^Chl^25^, and *S. albus* S12 transcriptomes was carried out with the aid of gene clustering analysis. The result showed that the expression pattern of all 1602 DEGs could be divided into 8 clusters (Figures 4A,B). Among these 8 clusters, cluster 7 where gene expression is continuously up-regulated was selected as an analytical model based on *p*-value significance (Figure S6) and its correspondence with salinomycin titer. Cluster of Orthologous Groups analysis (COG Analysis) of the 160 DEGs in cluster 7 was performed to see which metabolic pathways were associated with salinomycin biosynthesis. As shown in Figure 5, it was found that the relevant differential pathways affecting salinomycin biosynthesis were mainly related to butanoate metabolism, starch and sucrose metabolism, glyoxylate metabolism. In short, these metabolic pathways were more active in high-yield salinomycin production strain. Butanoate metabolism is a crucial source of salinomycin synthesis precursor ethylmalonyl-CoA, and glyoxylate metabolism might be involved in the arrangement of TCA cycles. Similarly, Lu et al. (2018) *also found that Aspergillus niger* with more enzyme production under limited oxygen environment had an increased flux of glyoxylate metabolism.

### DEGS Related to Secondary Metabolism Gene Clusters

**Figure 6:**
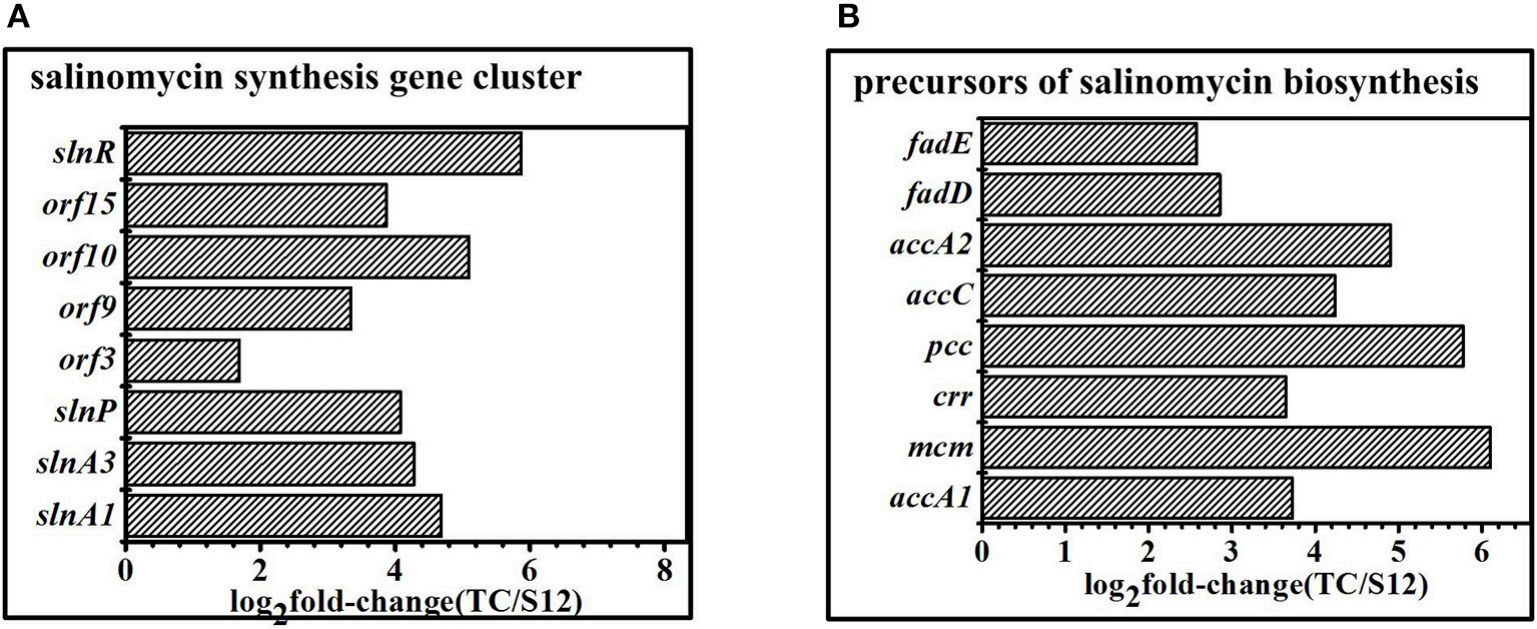
DEGS related to secondary metabolism gene clusters. **(A)** Expression of genes in salinomycin biosynthesis gene cluster. **(B)** Expression of genes related to precursors of salinomycin biosynthesis. Histograms show the abundance of the transcripts with significant variations (*p*-value<0.05) discussed in the text. Abundance values are shown in log_2_ fold-change (TK/S12). For detail genes expression (see Table S7).

The salinomycin biosynthesis gene cluster was identified, and the mechanism for salinomycin biosynthesis was proposed by different researchers in the previous studies (Yurkovich et al., 2011; Jiang et al., 2012, 2015). When compared with the original strain S12, expression of genes related to salinomycin biosynthesis gene cluster showed significant difference with Tet^30^Chl^25^ strain. Specifically, the positive pathway-specific regulator *sln*R (*SLNWT_0261*) and *pks* genes (*slnA1, slnA3*) were highly up-regulated in corresponding with the high production of salinomycin in mutant strain Tet^30^Chl^25^. Moreover, the transcription regulator genes (*orf* 3, *orf* 9, *orf* 10, *orf* 15) (Jiang et al., 2012; Zhu et al., 2017) were also highly up-regulated in Tet^30^Chl^25^ strain (Figure 6A). In other words, transcription of genes in the salinomycin biosynthesis gene cluster was enhanced in a high yield of salinomycin mutant.

Malonyl-CoA (M-CoAs), methylmalonyl-CoA (MM-CoAs), ethylmalonyl-CoA (EM-CoAs) are considered as the main precursors of salinomycin biosynthesis (Lu et al., 2016). Furthermore, transcripts of these genes (*accA1, accA2, accC, pcc, mcm, crr*) involved in precursors biosynthesis were highly up-regulated in high-yield strainTet^30^Chl^25^, but there were no significant changes in low-yield mutant strain TK (Figure 6B), which suggested that the resistance screening and ARTP increased anabolic flux through salinomycin precursors synthesis pathway in mutant strain Tet^30^Chl^25^.

### Overexpression of Potential Key Genes to Improve Salinomycin Production in *S. albus*

As mentioned above, we found that transcription level of genes *orf* 3, *orf* 9, *orf* 10, and *orf* 15 in the salinomycin biosynthesis gene cluster and their related genes in the precursor's biosynthesis pathway were more active in high-yield salinomycin producing strain Tet^30^Chl^25^. However, more research is required to explore their roles in the process of salinomycin biosynthesis. Therefore, genes including *orf* 3, *orf* 9, *orf* 10, and *orf* 15 located in the salinomycin biosynthesis gene cluster and *crr* gene, encoding crotonyl-CoA reductase in precursor's synthesis pathway were selected to be overexpressed in wild type strain DSM41398 or an industrial strain S12.

**Figure 7:**
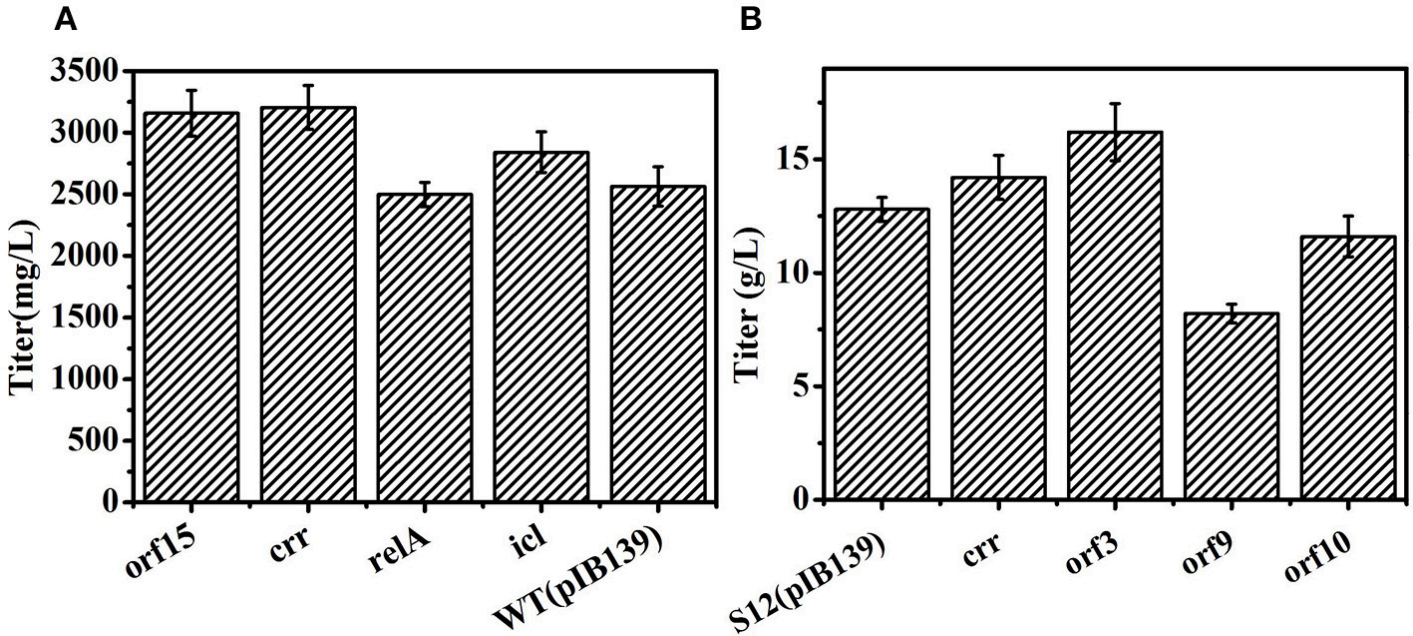
Overexpression of related genes to improve salinomycin in *S. albus*. **(A)** Overexpression of genes *orf15, crr, relA, icl* in wild type strain DSM41398. **(B)** Overexpression of genes *crr, orf3, orf9, orf10* in the industrial strain S12. Error bars indicate standard error of the mean.

As shown in Figures 7A,B, overexpression genes of *orf* 3, *orf* 15, and *crr* enhanced salinomycin production in DSM41398 or S12, indicating that these genes were involved in salinomycin biosynthesis and were insufficient during salinomycin biosynthesis in low yield strains. However, overexpression genes of *orf* 9 and *orf* 10 caused a decrease in salinomycin production of the industrial strain S12. Besides, the pathway of glyoxylate metabolism was closely related to salinomycin synthesis (Figure 5), and gene *relA* encoding enzyme catalyzing (p) ppGpp synthesis was significantly up-regulated in high-yield salinomycin producer mutant Tet^30^Chl^25^. As such, gene *rel*A and gene *SLNWT_6749* encoding isocitrate lyase (*icl*) in glyoxylate metabolism were also overexpressed in wild type strain DSM41398. As compared with wild type strain DSM41398, the results show no obvious differences in salinomycin production of mutant strain with overexpressed gene *relA*. It inferred that the regulation of salinomycin synthesis was very complicated, and a simply increased expression of *relA* could not have directly resulted in accumulation of (p) ppGpp. Whereas, the titer of salinomycin production was increased by 11% in mutant strain with the overexpressed gene *icl*, verifying that enhanced flux of glyoxylate metabolism could lead to an increase in salinomycin biosynthesis (Figure 7A). Therefore, *S. albus* mutated by ARTP and antibiotic resistance screening can generate a great diversity of mutants. Moreover, the mutated genes, regardless of being high-yield or low-yield, are mainly related to precursor's formation pathways and salinomycin biosynthesis pathway.

## Discussion

### Highly Efficient Breeding Method Development for Salinomycin Production

In this study, ribosome engineering, atmospheric and room temperature plasma (ARTP) and high-throughput screening (HTS) are extensively applied to screening of high-yield strains of salinomycin. The developed breeding strategy for salinomycin strain is presented in Figure 2. This strategy has several advantages over previous methods (Figure 2). Firstly, it is a precise and visualized method in which agar block diffusion method has high accuracy (over 70%) when selecting target mutants in the breeding process. Besides, it is very simple to pick high-yield mutants of salinomycin with larger diameter of inhibition zone using the naked eye. Secondly, the developed breeding strategy is low labor-consumption and greatly shortened the screening duration by replacing 24-well plate fermentation screening with agar block diffusion method screening in preliminary screening. At the same time, the optimized screening duration was reduced by 18 days.

A high-yield salinomycin mutant Tet^30^Chl^25^ with tetracycline and chloramphenicol resistance was finally obtained by high-throughput screening from 10^6^ to 10^8^ mutants. In the shake flask culture, mutant Tet^30^Chl^25^ was hereditarily stable and the yield of salinomycin was increased by 1.1 times, as compared to 16,306 mg/L produced by the industrial strain S12 (Figure 3D). This indicated that the combined method is an effective strategy for strain breeding. However, the yield of mutant was much lower than the yield of commercial fermentation (100 g/L), which is due to the low yield of the initial strain (Luhavaya et al., 2015).

Furthermore, this strategy can generate a diversity of mutants at gene level. The gene transcripts of mutants Tet^30^Chl^25^ and TK show a great difference with the original strain S12 (Figure S5), after treatment by APTP and resistance screening. As shown in Figure 5, metabolic pathways, including butanoate metabolism, starch and sucrose metabolism, and glyoxylate metabolism are found to be closely related to salinomycin biosynthesis. Thus, we verify that enhanced flux of glyoxylate metabolism via overexpressed gene *icl* can lead to an increase in salinomycin biosynthesis. Besides, transcriptional regulation genes like *orf* 3 and *orf* 15, located in salinomycin biosynthesis clusters, and *crr* gene are positively linked with salinomycin production, and their mutation possibly resulted in enhanced salinomycin synthesis.

### Proposed Mechanism of ARTP Mutagenesis and Resistance Screening to Improve Salinomycin Production

Generally, ARTP treatment can generate random mutagenesis at the gene level, leading to DNA damages such as missense mutation, deletion or nucleotide frameshift mutation (Fang et al., 2013; Li et al., 2014; Zhang et al., 2014). Ochi et al. (2003) found that genes encoding ribosomal protein, stringent response and/or RNA polymerases played a central role in ribosome engineering. In this work, we also found that genes related to synthesis of ribosomal protein, stringent response and RNA polymerases show obvious variations between mutants and the initial strain S12 based on their transcriptomic dataset.

### Genes for Ribosomal Protein

**Figure 8:**
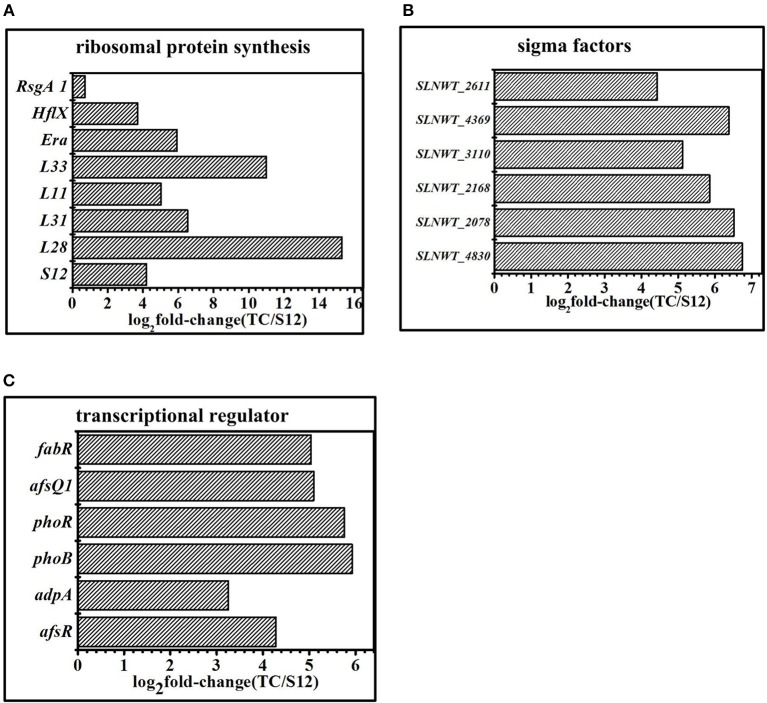
Transcriptomic analysis of the mutants and the original strain S12 at the 5 days in fermentation medium. **(A)** Expression of genes related to ribosomal protein. **(B)** Expression of genes related to sigma factors. **(C)** Expression of genes related to transcriptional regulator. Histograms show the abundance of the transcripts with significant variations (*p*-value<0.05) discussed in the text. Abundance values are shown in log_2_ fold-change (TK/S12). For detail genes expression (see Table S7).

Compared with the original strain S12, transcripts of ribosomal proteins exhibited obvious differences among mutant strain TK and Tet^30^Chl^25^. Gene expression of ribosomal protein S12 (*SLNWT_4578*), L28(*SLNWT_2930*), L31(*SLNWT_1937*), L33(*SLNWT_2931*), L11(*SLNWT_4559)* were significantly up-regulated in high-yield salinomycin strain Tet^30^Chl^25^ (Figure 8A). GTPase was thought to functionally promote ribosome assembly and to change the pools of ATP/GTP (Corrigan et al., 2016). The transcription of GTPases *Era*(*SLNWT_5342*), *HflX*(*SLNWT_1473*), and *RsgA1* (SLNWT_0717) increased as these were overexpressed in Tet^30^Chl^25^ strain (Figure 8A). In addition, expression of gene *relA*, encoding an enzyme that catalyzes the synthesis of guanosine pentaphosphate/tetraphosphate ((p)ppGpp), responsible for the stringent response (Steinchen and Bange, 2016), was up-regulated in both Tet^30^Chl^25^ and TK strains. These results indicated that ARTP mutagenesis and resistance screening process could generate mutants with a gene mutation of ribosomal proteins and stringent response.

### Genes for Sigma Factors and Transcriptional Factors

Sigma factors and transcriptional factors played a crucial role in bacterial signaling transduction systems. The expression of sigma factor genes (*sigB, sigQ, hrdD, bldN, sigE*) in mutants and wild type strains is shown in Figure 8B. Extracytoplasmic sigma factor (ECF sigma factor) genes were activated by stress conditions like ultraviolet radiation or resistance selection and showed a higher level of expression in Tet^30^Chl^25^ strains. These genes were *sigE* (*SLNWT_2168*), which played an essential role ensuring the integrity of the bacterial cell envelope (Hayden and Ades, 2008); *sigB* (*SLNWT_2611, SLNWT_3110, SLNWT_2078)*, related to pressure response protein, and *sigQ* (*SLNWT_4830*), a target of two-component system (TCSs) afsQ1/Q2 (Wang et al., 2013). Besides, transcriptional factors clearly appeared up-regulated in strain Tet^30^Chl^25^ (Figure 8C). Among these, transcription regulators of the two-component systems a*fsQ1/Q2* are known for positively regulating the secondary metabolism (Wang et al., 2013). *glnR* is an essential gene that controls nitrogen source in Streptomyces (Yao et al., 2014) and other transcriptional regulators, *phoR/B* and *afs*R, respond to regulation of phosphate metabolism in *Streptomyces*.

**Figure 9:**
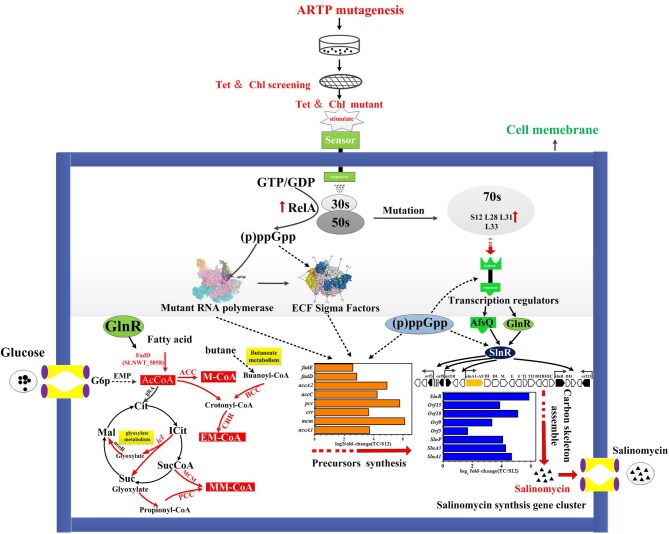
Proposed mechanism of ARTP mutagenesis and resistance screening to improve salinomycin production: 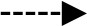 upregulated; 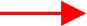 directly control; 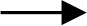 indirectly control; or unclear.

A tentative regulatory model of high-yield salinomycin mutant Tet^30^Chl^25^ after ARTP treatment and resistance screening is proposed as likely shown in Figure 9. This model involved three aspects of the signal transduction process. Firstly, stringent response was activated along with accumulation of (p)ppGpp and mutation of ribosomal proteins. The stress signal caused by ARTP mutagenesis and resistance screening was crossed through the cell membrane, accompanied by a series of signal transduction reactions, which included activation of gene *relA* and accumulation of (p)ppGpp. Later, (p)ppGpp might have induced direct regulation of ribosomal protein promoters (Lemke et al., 2011). Besides, the binding targets of tetracycline and chloramphenicol were ribosomal 30s subunit and the A-site crevice of 50S subunit, respectively (Jenner et al., 2013; Lin et al., 2018), this interaction may also result in changing the expression of ribosomal proteins. Thus, the expression level of genes in Tet^30^Chl^25^ strain encoding ribosomal proteins L11, L31, L28, L33, and S12, were evidentially increased as shown in Figure 8A. Particularly, ribosome protein L11 is the target site of protein RelA and also involved in the synthesis of (p)ppGpp (Lino et al., 2018). In another report, the mutation of protein S12 was proven to result in preferential transcription of specific genes, such as *act*II-ORF4 an *red*D during the late growth phase of *S. coelicolor* (Hosaka et al., 2004).

Secondly, accumulation of (p)ppGpp and mutational ribosomal protein may further activate the signal transduction system, including activation of sigma factors and transcriptional factors. Accumulation of (p)ppGpp under stringent stress condition also exerted influence alterations in gene expression owing to changes in RNA polymerase (RNAP) activity Many genetic studies of *E. coli* have suggested that RNA polymerase (RNAP) is the target of (p)ppGpp regulation (Ding and Gross, 1988). In the case of *E. coli* RNAP, (p)ppGpp directly interacted with RNAP to destabilize the short lived open complexes (Paul et al., 2005). Mutant RNA polymers and (p)ppGpp in turn activated targeted signal transduction genes like transcription factors and ECF sigma factors (sigE sigB sigQ), etc. (Inaoka et al., 2004; Alessandra and Ades, 2006).

Thirdly, the activated transcriptional regulators and ECF sigma factor further affected the expression of genes in salinomycin biosynthesis gene cluster (*slnA1-A9, slnR, orf3, orf15*) (Figure 6A), salinomycin precursors synthesis pathway (*accA1, accA2, pcc, mcm, crr*) (Figure 6B) and antibiotic synthesis-related pathways like butanoate metabolism, starch and sucrose metabolism, and glyoxylate metabolism (Figure 5). Moreover, transcriptional regulators TCSs *afs*Q1/Q2 were supposed to positively regulate the production of ACT and RED in *S. coelicolor* (Wang et al., 2013). In this study, we found that both protein AfsQ1 and GlnR could directly bind to the promoter of the positive pathway-specific regulator SlnR, and GlnR could also directly bind to the promoter of *fadD* (*SLNWT_5058*, coding a key enzyme in fatty acid metabolism) (Figure S7). Extensive prior research revealed that ECF sigma factors were involved in secondary metabolism, and these are capable of directly binding the promoters of genes related to antibiotic synthesis, aerial mycelium differentiation and sporulation (Costanzo et al., 2010; Feng et al., 2011; Wang et al., 2013; Luo et al., 2014; López-García et al., 2018).

However, the proposed mechanisms of ARTP and ribosome engineering for improving salinomycin production were established based on transcriptomes analysis but they still remain ambiguous. The direct or indirect interactions among ribosomal protein, string response, two component system and sigma factors are complex and unclear, and further research is required to reveal these interactions.

## Data Availability

Raw data are available on the Gene Expression Omnibus database (accession GSE133580).

## Author Contributions

All authors read and approved the manuscript. All authors contributed significantly to the work. MG and KZ conceived the project. MG, KZ, and YZ designed the experiments and analyzed results. KZ wrote the manuscript with the help of AM, MG, YZ, and JC. KZ performed experiments and supported by YD and ZC.

### Conflict of Interest Statement

ZC was employed by the company Zhejiang Biok Biology Co., Ltd. The remaining authors declare that the research was conducted in the absence of any commercial or financial relationships that could be construed as a potential conflict of interest.
