# Supplementary material for: Combinatorial Effect of ARTP Mutagenesis and Ribosome Engineering on an Industrial Strain of Streptomyces albus S12 for Enhanced Biosynthesis of Salinomycin
Source: Front Bioeng Biotechnol. 2019 Sep 3;7:212. doi: 10.3389/fbioe.2019.00212 (PMC6733881; doi:10.3389/fbioe.2019.00212)
Supplement: Supplementary file 1 [file Table_1.docx]

**Supplementary material**

**Tables**

**Table S_1_**. The single antibiotic concentration of plate

| **Antibiotics\treatment number** | 1 | 2 | 3 | 4 | 5 | 6 | 7 | 8 | 9 |
| --- | --- | --- | --- | --- | --- | --- | --- | --- | --- |
| Kanamycin  （μg/mL） | 1 | 1.3 | 1.5 | 1.8 | 2.0 | 2.3 | 2.5 | 2.8 | 3.0 |
| Tetracycline  （μg/mL） | 20 | 23 | 25 | 28 | 30 | 33 | 35 | 38 | 40 |
| Chloromycetin  （μg/mL） | 15 | 18 | 20 | 23 | 25 | 28 | 30 | 33 | 35 |

**Table S_2_**. The Kan-Tet antibiotic concentration of plate

| **Antibiotics\treatment number** | 1 | 2 | 3 | 4 | 5 | 6 | 7 | 8 | 9 |
| --- | --- | --- | --- | --- | --- | --- | --- | --- | --- |
| Kanamycin  （μg/mL） | 1 | 1.3 | 1.5 | 1.8 | 2.0 | 2.3 | 2.5 | 2.8 | 3.0 |
| Tetracycline  （μg/mL） | 20 | 23 | 25 | 28 | 30 | 33 | 35 | 38 | 40 |

**Table S_3_**. The Kan-Chl antibiotic concentration of plate

| **Antibiotics\treatment number** | 1 | 2 | 3 | 4 | 5 | 6 | 7 | 8 | 9 |
| --- | --- | --- | --- | --- | --- | --- | --- | --- | --- |
| Kanamycin  （μg/mL） | 1 | 1.3 | 1.5 | 1.8 | 2.0 | 2.3 | 2.5 | 2.8 | 3.0 |
| Chloromycetin  （μg/mL） | 15 | 18 | 20 | 23 | 25 | 28 | 30 | 33 | 35 |

**Table S_4_**. The Tet-Chl antibiotic concentration of plate

| **Antibiotics\treatment number** | 1 | 2 | 3 | 4 | 5 | 6 | 7 | 8 | 9 |
| --- | --- | --- | --- | --- | --- | --- | --- | --- | --- |
| Tetracycline  （μg/mL） | 20 | 23 | 25 | 28 | 30 | 33 | 35 | 38 | 40 |
| Chloromycetin  （μg/mL） | 15 | 18 | 20 | 23 | 25 | 28 | 30 | 33 | 35 |

**Table S_5_**. The Kan-Tet-Chl antibiotic concentration of plate

| **Antibiotics\treatment number** | 1 | 2 | 3 | 4 | 5 | 6 | 7 | 8 | 9 |
| --- | --- | --- | --- | --- | --- | --- | --- | --- | --- |
| Kanamycin  （μg/mL） | 1 | 1.3 | 1.5 | 1.8 | 2.0 | 2.3 | 2.5 | 2.8 | 3.0 |
| Tetracycline  （μg/mL） | 20 | 23 | 25 | 28 | 30 | 33 | 35 | 38 | 40 |
| Chloromycetin  （μg/mL） | 15 | 18 | 20 | 23 | 25 | 28 | 30 | 33 | 35 |

Table **S_6_**. Diameter of inhibitory zone with indicating bacterial cells grown in different pre-cultivation modes.

| Density of indicating bacterial（%） | Diameter of inhibitory zone（mm） | |
| --- | --- | --- |
|  | a | b |
| 0 | 0 | 0 |
| 0.05 | 16.72±0.25 | 15.83±0.34 |
| 0.1 | 19.78±0.58 | 18.10±0.47 |
| 0.15 | 18.89±0.33 | 16.77±0.20 |
| 0.2 | 15.66±0.37 | 12.95±0.41 |

a .,pre-cultivation at 4℃ ,b ., cultivation directly at 30 ℃

**Table S_7_.** Abundance values of transcripts showing signiﬁcant variations (p-value < 0.05) between the mutant TC,TK and the original strain S12 quantiﬁed in fermentation cultures (5 days), and grouped into functional categories.

| **Genes related to ribosome synthesis** | | | |
| --- | --- | --- | --- |
| Code | Product | log_2_fold-change(TC/S12) | log_2_ fold-change(TK/S12) |
| SLNWT_4578 | 30S ribosomal protein S12 | 4.199 | 0.323 |
| SLNWT_2930 | 50S ribosomal protein L28 | 15.284 | 2.048 |
| SLNWT_1937 | 50S ribosomal protein L31 | 6.534 | 1.210 |
| SLNWT_2931 | 50S ribosomal protein L33 | 11.0 | -0.398 |
| SLNWT_4559 | 50S ribosomal protein L11 | 5.029 | 0.551 |
| SLNWT_5342 | Era | 5.925 | 1.299 |
| SLNWT_1473 | HflX | 3.682 | 0.167 |
| SLNWT_0717 | RsgA 1 | 0.717 | -0.799 |
| SLNWT_6425 | RelA | 5.330526 | 0.826 |
| **Genes related to precursors biosynthesis** | | | |
| SLNWT_0171 | ACCA1 | 3.727 | -3.411 |
| SLNWT_1888 | MCM | 6.098 | 0.535 |
| SLNWT_0464 | CRR | 3.647 | -2.039 |
| SLNWT_4849 | PCC | 5.773 | 0.544 |
| SLNWT_2336 | ACCC | 4.238 | 0.441 |
| SLNWT_4844 | ACCA2 | 4.901 | 0.055 |
| SLNWT_5058 | FadD | 2.853 | 0.505 |
| SLNWT_4686 | FadE | 2.578 | -0.951 |
| **Genes related to salinomycin synthesis clusters** | | | |
| SLNWT_0280 | SlnA1 | 4.686 | -0.798 |
| SLNWT_0278 | SlnA3 | 4.277 | -0.463 |
| SLNWT_0282 | SlnP | 4.077 | -2.098 |
| SLNWT_0267 | Cytochrome P450 | 7.722 | -0.282 |
| SLNWT_0290 | Orf3 | 1.689 | 0.634 |
| SLNWT_0284 | Orf9 | 3.345 | 0.230 |
| SLNWT_0283 | Orf10 | 5.093 | -0.536 |
| SLNWT_0255 | Orf15 | 3.871 | 0.412 |
| SLNWT_0261 | SlnR | 5.869 | -0.312 |
| **Genes related to sigma Factor synthesis** | | | |
| SLNWT_4830 | SigQ | 6.746 | -0.707 |
| SLNWT_2078 | SigB | 6.516 | 1.448 |
| SLNWT_2168 | SigE | 5.855 | 1.707 |
| SLNWT_3110 | SigB | 5.122 | -0.474 |
| SLNWT_4369 | HrdD | 6.384 | 0.984 |
| SLNWT_2611 | SigB | 4.427 | 2.070 |
| **Genes related to transcriptional regulator** | | | |
| SLNWT_4331 | afsR | 4.283 | 0.389 |
| SLNWT_2349 | adpA | 3.249 | 1.878 |
| SLNWT_4132 | phoB | 5.926 | -0.241 |
| SLNWT_4131 | phoR | 5.756 | -0.282 |
| SLNWT_4829 | afsQ1 | 5.096 | 1.299 |
| SLNWT_1769 | FabR | 5.042 | -0.088 |

**The genes discussed in the table are indicated.**

**Figures**


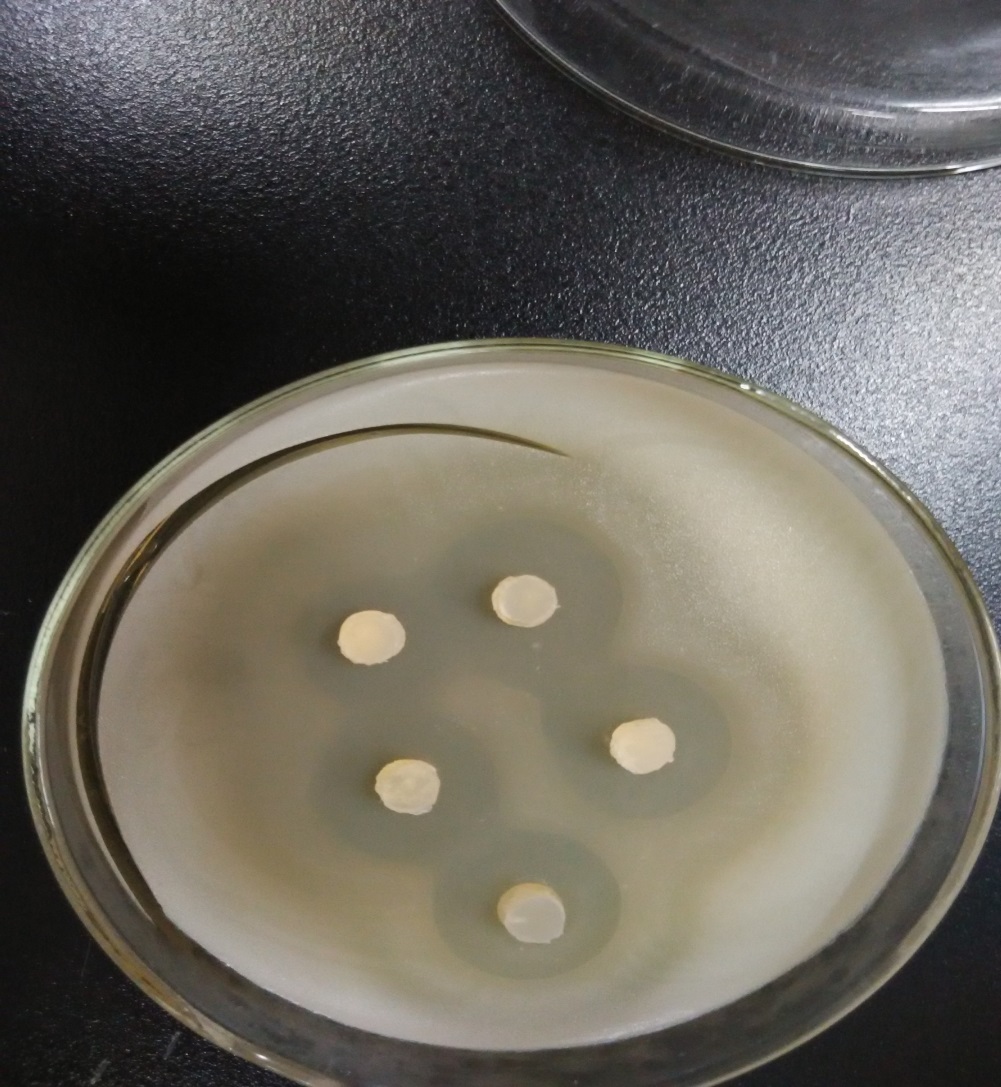


**Fig. S_1_.** Inhibitory zone of salinomycin against bacterial cells grown on agar plate


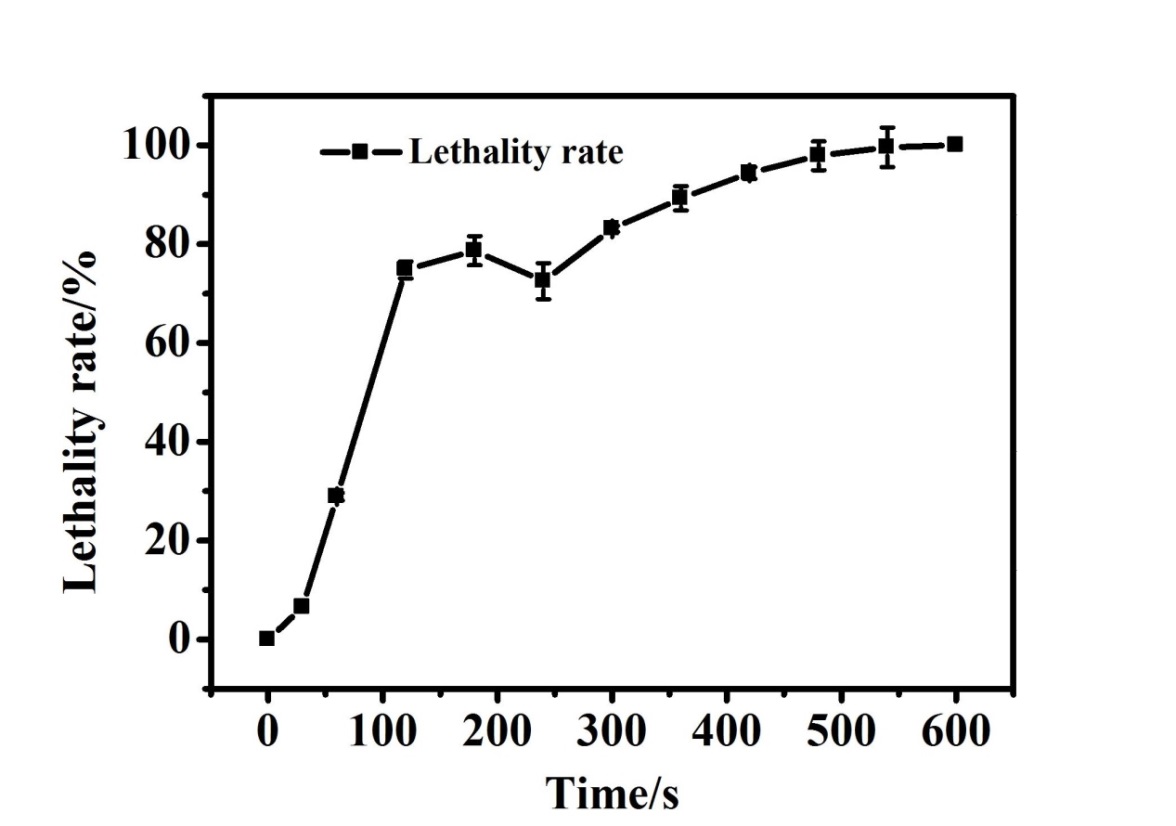


**Fig. S_2_.** Lethality curve of *S. albus* after ARTP treatment





**Fig. S_3_.** Effect of spore population of *Streptomyces albus* on diameter of inhibitory zone


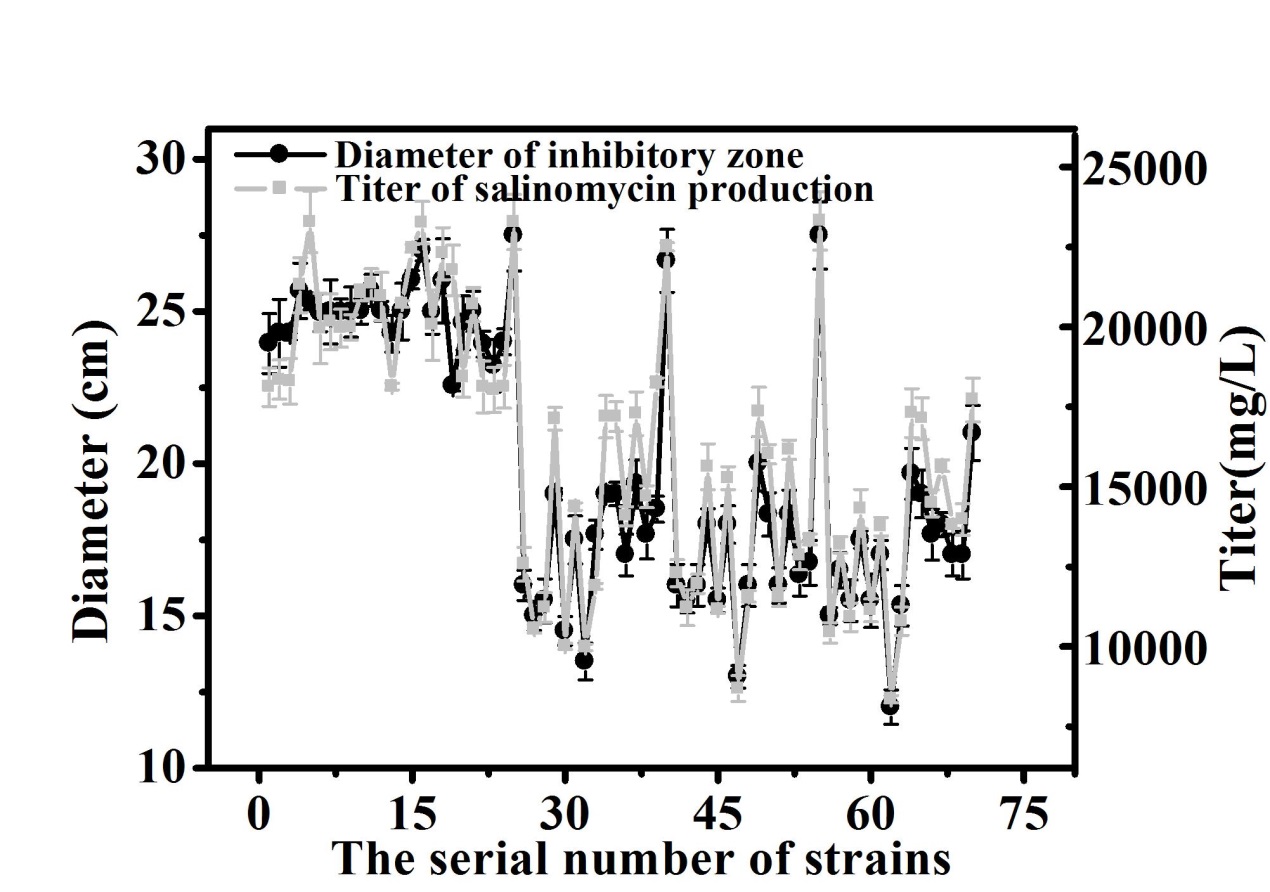


**Fig. S_4_.** Correlation between inhibitory zone and titer of salinomycin


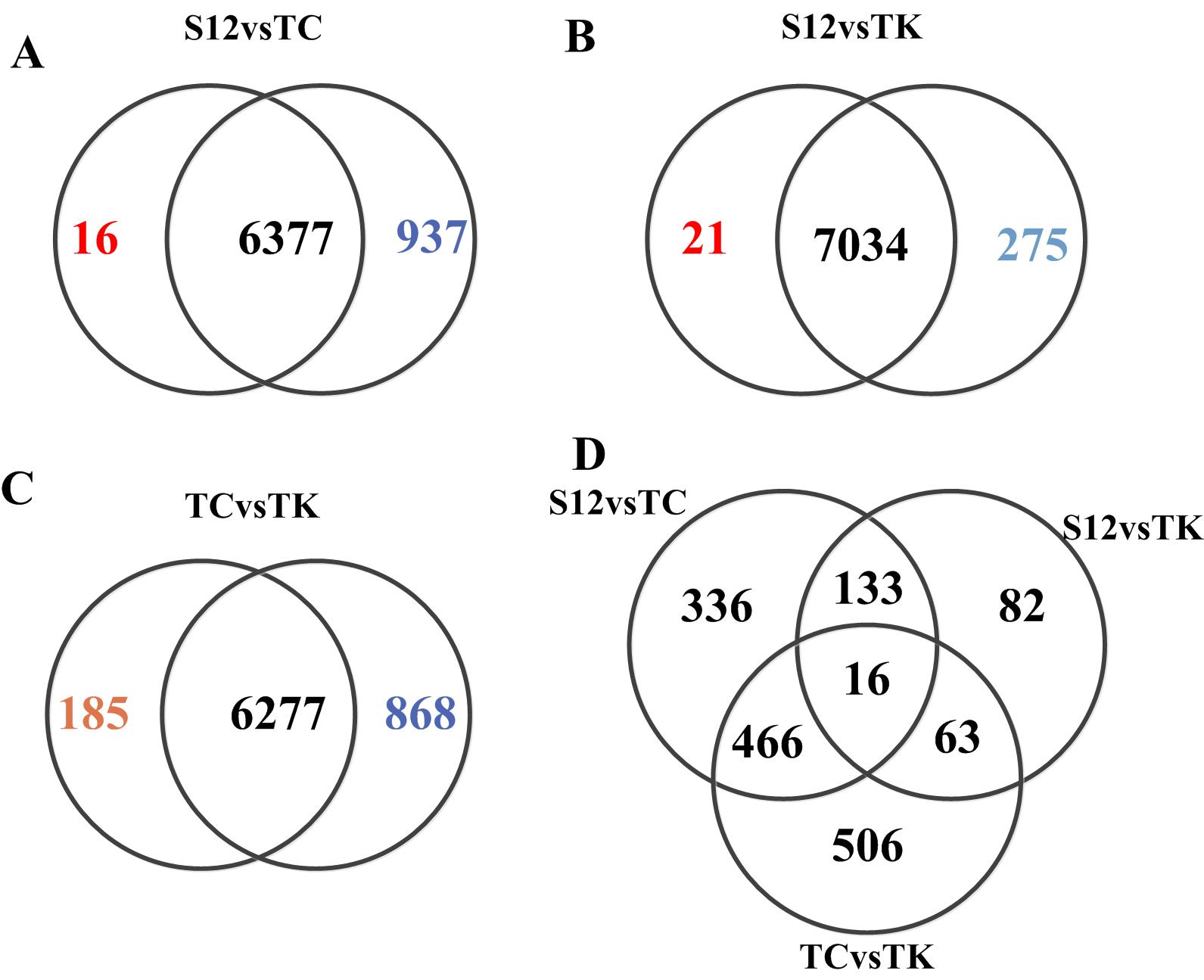


**Fig. S_5_.** Differentially expressed genes(DEGs) (fold change>2 or fold change<0.5, signiﬁcation level p < 0.05, FDR<0.05) between the mutant strain TK, TC and the initial strain S12. (A). Venn diagram shows transcripts with signiﬁcant up-regulation in the S12 strain(blue number) or the TC mutant(red number)and transcripts without signiﬁcant variations (black number). (B). Venn diagram shows transcripts with signiﬁcant up-regulation in the S12 strain(blue number) or the TK mutant(red number)and transcripts without signiﬁcant variations (black number). (C). Venn diagram shows transcripts with signiﬁcant up-regulation in the TK strain(blue number) or the TC mutant(red number)and transcripts without signiﬁcant variations (black number). (D). Venn diagram shows transcripts with signiﬁcant variations between the mutant strain TK, TC and the initial strain S12.

**Fig. S_6_.** Analysis of trend models with differentially expressed gene(DEGs). The number in the upper left corner of each trend represents the type number to which the trend belongs, the number in the lower left corner represents the p value which means the level of signifiance , the smaller the p value, the better the significance.


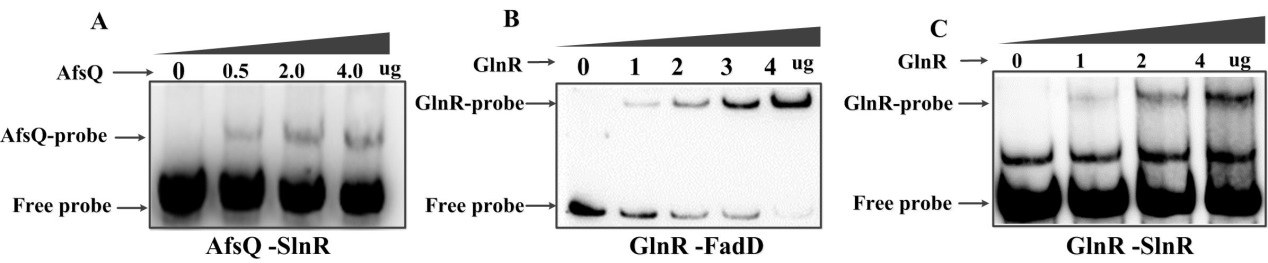


**Fig. S_7_.** EMSAs of His-AfsQ and His-GlnR protein with upstream promoter regions of slnR, fadD(SLNWT_5058). (A) EMSAs of His-AfsQ protein with upstream promoter regions of SlnR. (B) EMSAs of His-GlnR protein with upstream promoter regions of SLNWT_5058. (C) EMSAs of His-GlnR protein with upstream promoter regions of SlnR. The DNA probe (10 nM) was incubated with a protein concentration gradient (0, 0.5 and 4.0 ug).
